# Supplementary material for: The acidic intrinsically disordered region of the inflammatory mediator HMGB1 mediates fuzzy interactions with CXCL12
Source: Nat Commun. 2024 Feb 8;15:1201. doi: 10.1038/s41467-024-45505-7 (PMC10853541; doi:10.1038/s41467-024-45505-7)
Supplement: Supplementary file 1 — Supplementary Information [file 41467_2024_45505_MOESM1_ESM.pdf]

The acidic intrinsically disordered region of the inflammatory mediator HMGB1 mediates fuzzy interactions with CXCL12

Malisa Vittoria Mantonico[1,2], Federica De Leo[1,§], Giacomo Quilici[1], Liam Sean Colley[3,4], Francesco De Marchis[2,5], Massimo Crippa[5], Rosanna Mezzapelle[2,5], Tim Schulte[6], Chiara Zucchelli[1], Chiara Pastorello[1], Camilla Carmeno[1], Francesca Caprioglio[2,5], Stefano Ricagno[6,7], Gabriele Giachin[8], Michela Ghitti[1*], Marco Bianchi[2,5], Giovanna Musco[1*]

*e-mail: ghitti.michela@hsr.it; musco.giovanna@hsr.it

[1]Biomolecular NMR Laboratory, Division of Genetics and Cell Biology, IRCCS Ospedale San Raffaele, Milan, Italy

[2]School of Medicine, Università Vita e Salute-San Raffaele, Milan, Italy

[3]HMGBiotech S.r.l., 20133 Milan, Italy

[4]School of Medicine and Surgery, Università Milano-Bicocca, 20126 Milan, Italy

[5]Chromatin Dynamics Unit, Division of Genetics and Cell Biology, IRCCS Ospedale San Raffaele, Milan, Italy

[6]Institute of Molecular and Translational Cardiology, IRCCS Policlinico San Donato, Milan, Italy

[7]Department of Biosciences, Università degli Studi di Milano, Milan, Italy.

[8]Department of Chemical Sciences (DiSC), University of Padua, 35131 Padova, Italy.

[§]present addresses: Experimental Therapeutics Program, IFOM ETS - The AIRC Institute of Molecular Oncology and AIRC, Fondazione AIRC per la Ricerca sul Cancro ETS Milan, Italy

Supplementary Information

Supplementary Figures: page 3-20

Supplementary Tables: page 21-25

Supplementary Methods: page 26-28

Supplementary References: page 29

Supplementary Figures

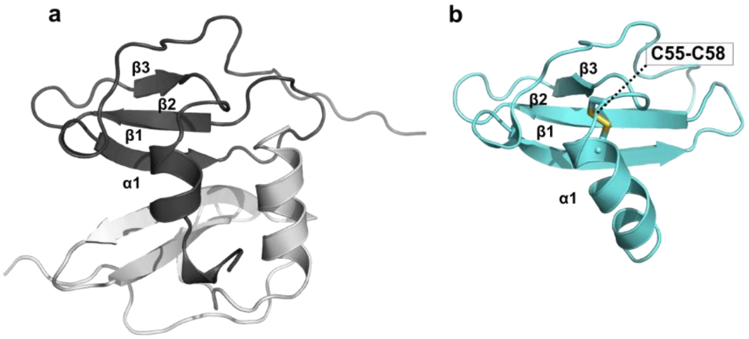

Supplementary Figure 1: Three dimensional structures of CXCL12 and CXCL12-LM. Cartoon representation of **a** the crystallographic structure of CXCL12 with two monomers in the asymmetric unit, (PDB code:1QG7 [https://doi.org/10.2210/pdb1QG7/pdb) and of **b** CXCL12-LM (PDB code: 2N55 [https://doi.org/10.2210/pdb2N55/pdb]), residues in position 55 and 58 have been mutated into cysteines, to generate a disulfide bond (shown in yellow sticks) that locks CXCL12 in a monomeric form.

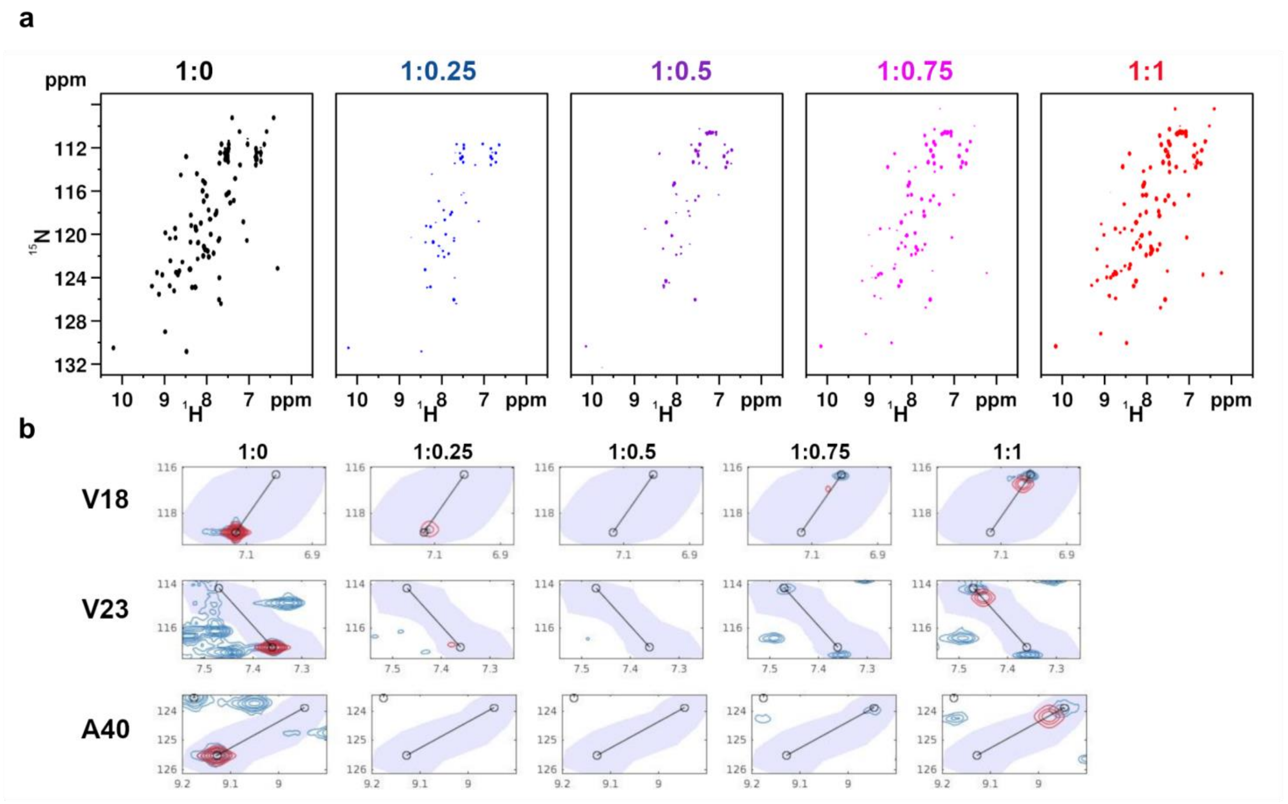

Supplementary Figure 2: Binding of Ac-pep to CXCL12. a ^{1}H-^{15}N HSQC spectra of ^{15}N CXCL12 (0.1 mM) in the presence of increasing concentrations of Ac-pep. Several peaks disappear at sub-stoichiometric ratio and reappear at equimolar ratio. **b** TITAN fits using single-site binding equation for CXCL12 resonance-specific trajectories as a function of Ac-pep. Original spectra are shown in blue contours, fitted spectra are shown in red contours. Three representative amide resonances are shown.

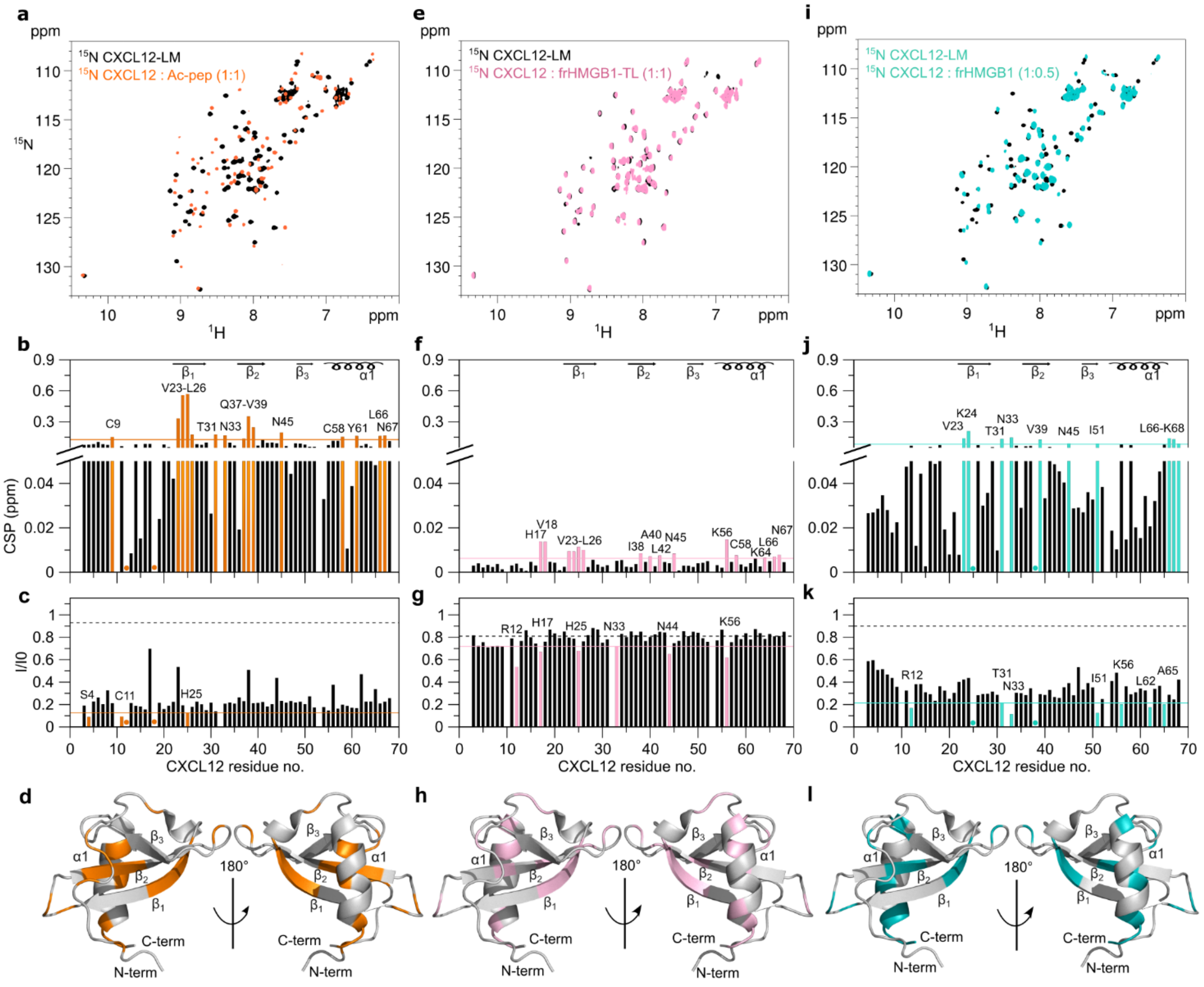

Supplementary Figure 3: The interaction of CXCL12-LM with Ac-pep, frHMGB1-TL and frHMGB1. a Superposition of ^{1}H-^{15}N HSQC spectra of ^{15}N CXCL12-LM (0.1 mM) without (black) and with (orange) Ac-pep (1:1). Bar graph showing residue-specific **b** chemical shift perturbations (CSPs) and **c** peak intensities ratios (I/I_0) of ^{15}N-labeled CXCL12-LM (0.1 mM) upon addition of Ac-pep (1:1). Residues with CSP > avg + σ_0 (corrected standard deviation, orange line) and with I/I_0 < avg - SD (standard deviation, orange line) are labeled and **d** represented in orange on CXCL12-LM (gray cartoon, pdb code: 2N55 [https://doi.org/10.2210/pdb2N55/pdb]). **e** Superposition of ^{1}H-^{15}N HSQC spectra of ^{15}N CXCL12-LM (0.1 mM) without (black) and with (pink) frHMGB1-TL (1:1). Bar graph showing residue-specific **f** CSPs and **g** *I/I0* of ^{15}N-labeled CXCL12-LM (0.1 mM) upon addition of frHMGB1-TL (1:1). Residues with CSP > avg + σ_0 (corrected standard deviation, pink line) and with I/I_0 < avg - SD (standard deviation, pink line) are labeled and **h** represented in pink on CXCL12-LM (gray cartoon, pdb code: 2N55 [https://doi.org/10.2210/pdb2N55/pdb]). **i** Superposition of ^{1}H-^{15}N HSQC spectra of ^{15}N CXCL12-LM (0.1 mM) without (black) and with (cyan) frHMGB1 (1:1). Bar graph showing residue-specific **j** CSPs and **k** I/I_0 of ^{15}N-labeled CXCL12-LM (0.1 mM) upon addition of frHMGB1 (1:1). Residues with CSP > avg + σ_0 (corrected standard deviation, cyan line) and with I/I_0 < avg - SD (standard deviation, cyan line) are labeled and **l** represented in cyan on CXCL12-LM (gray cartoon, pdb code: 2N55 [https://doi.org/10.2210/pdb2N55/pdb]). In the bar graphs α-helices and β-strands are schematically represented on the top, missing residues are prolines or are absent because of exchange with the solvent, dots indicate resonances that disappeared upon binding; the dashed black line indicates the peak intensity decrease due to the titration dilution effect.

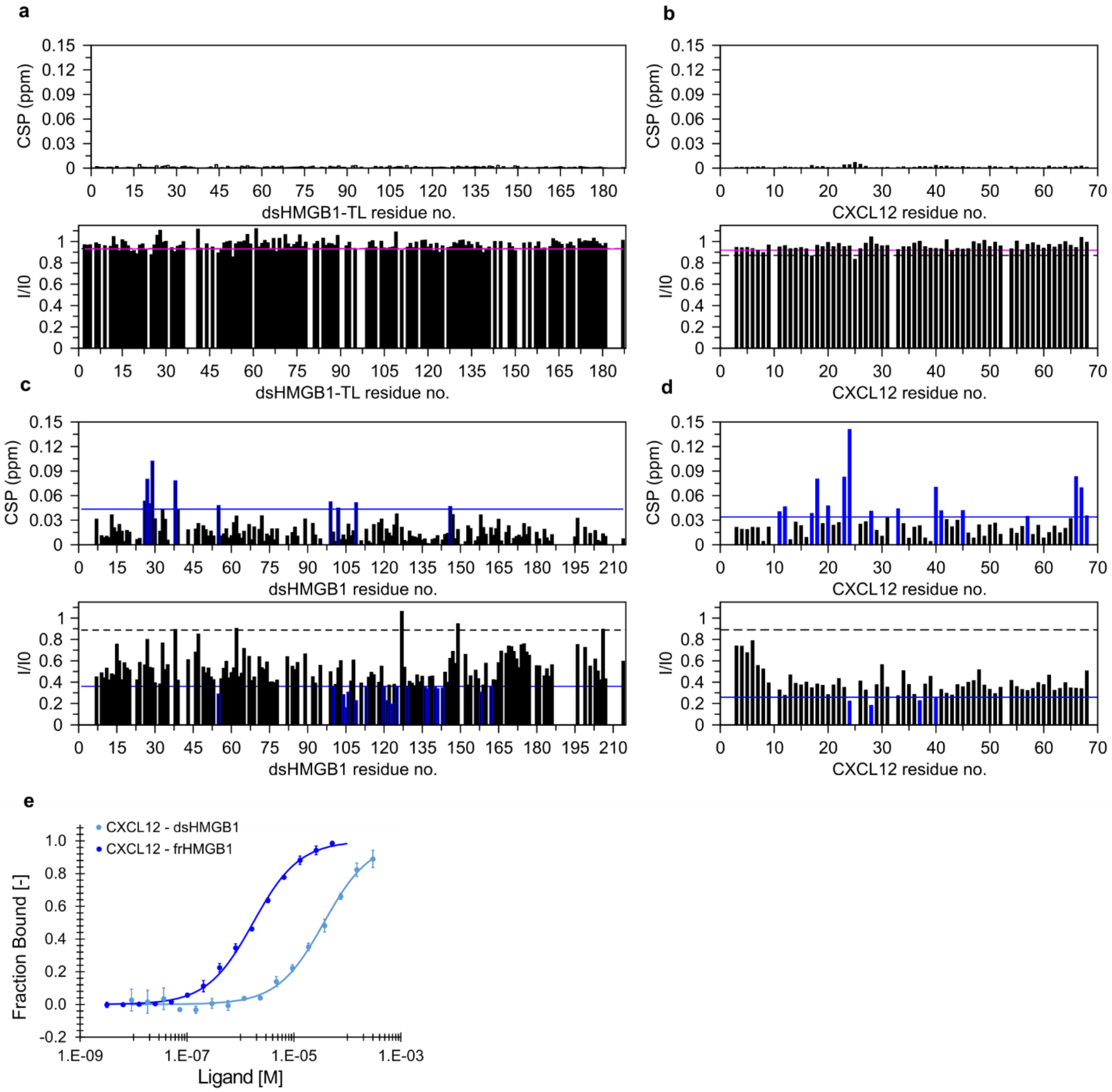

Supplementary Figure 4: Binding assays between dsHMGB1-TL, dsHMGB1 and CXCL12. Bar graphs showing **a** residue-specific chemical shift perturbations (CSPs) and peak intensity ratios (I/I_0) of ^{15}N-labeled dsHMGB1-TL (0.1 mM) upon addition of CXCL12 (1:1). Bar graphs showing **b** residue-specific CSPs and I/I_0 of ^{15}N-labeled CXCL12 (0.1 mM) upon addition of dsHMGB1-TL (1:1). In **a,b** the pink line and the black dashed lines represent the avg - SD (standard deviation) and peak intensity decrease due to the titration dilution effect, respectively. Bar graphs showing **c** residue-specific CSPs and I/I_0 of ^{15}N-labeled dsHMGB1 (0.1 mM) upon addition of CXCL12 (1:1). Bar graphs showing **d** residue-specific CSPs and I/I_0 of ^{15}N-labeled CXCL12 (0.1 mM) upon addition of dsHMGB1 (1:0.5). In **c,d** residues with CSP > avg + σ_0 (corrected standard deviation, blue line) and with I/I_0 < avg - SD (standard deviation, blue line) are represented in blue; missing residues are prolines or are absent because of exchange with the solvent; the dashed black line indicates the peak intensity decrease due to the titration dilution effect. **e** Normalized variation of MST signal of fluorescently labeled CXCL12 in the presence of frHMGB1 (blue) (Kd=1.7 ± 0.2 µM) and of dsHMGB1 (light blue) (Kd=37.3 ± 3.3 µM), n = 3 independent replicates. Data are presented as mean ± SD. NMR and MST titrations have been performed at pH 6, 20 mM NaCl, 20 mM phosphate buffer.

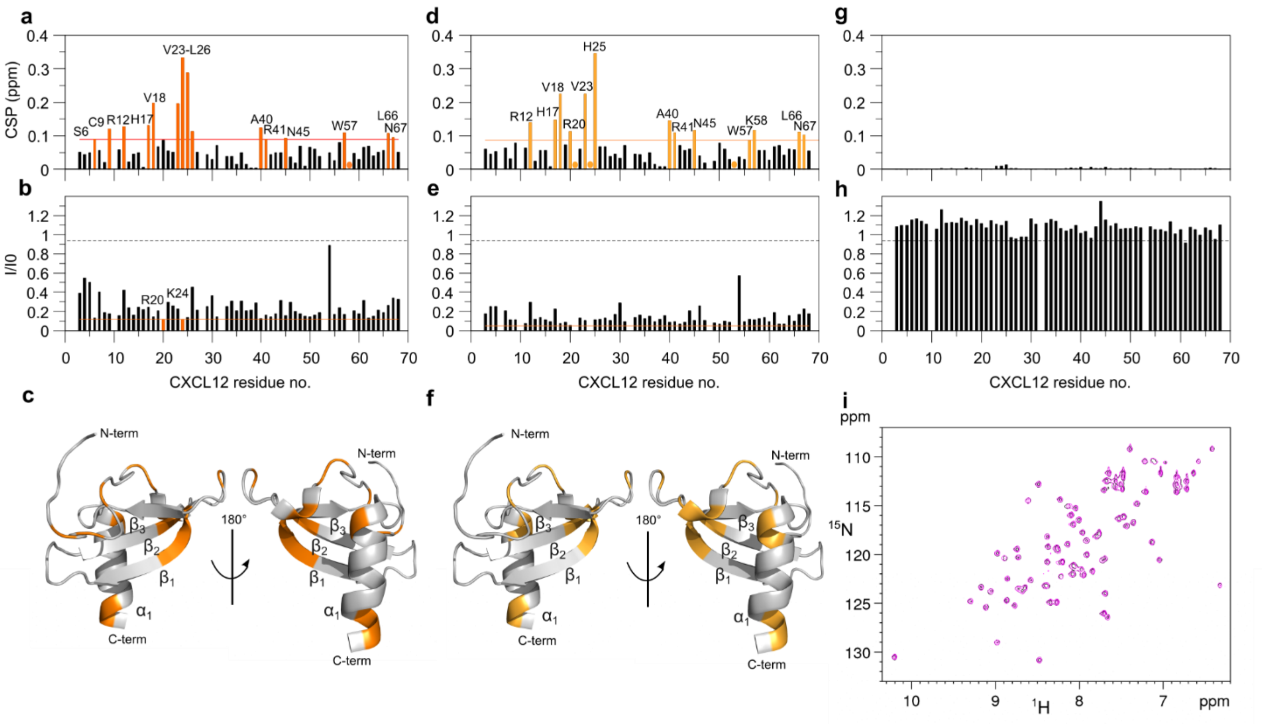

Supplementary Figure 5: Ac-pep interacts with CXCL12, while the arginine/lysine rich-peptide derived from HMGB1 aberrant form does not. a Bar graph showing residue-specific chemical shift perturbations (CSP) and **b** peak intensities ratios (*I/I_0*) of ^{15}N-labeled CXCL12 (0.1 mM) upon addition (Ac-pep$_{185\text{-}195}$) (1:3). Residues with CSP > avg + σ_0 (corrected standard deviation, orange line) and with *I/I_0* < avg - SD (standard deviation, orange line) are labeled and **c** shown in orange on CXCL12 (gray cartoon, pdb code: 2KEE [https://doi.org/10.2210/pdb2KEE/pdb]). Bar graph showing **d** residue-specific CSP and **e** *I/I_0* of ^{15}N-labeled CXCL12 (0.1 mM) upon addition (1:3) of (Ac-pep$_{204\text{-}214}$). Residues with CSP > avg + σ_0 (corrected standard deviation, light orange line) with *I/I_0* < avg – SD (standard deviation, orange line) are labeled and **f** shown in light orange on CXCL12 (gray cartoon, pdb code: 2KEE [https://doi.org/10.2210/pdb2KEE/pdb]). Bar graph showing **g** residue-specific CSPs and **h** *I/I_0* of ^{15}N-labeled CXCL12 (0.1 mM) upon addition (1:3) of arginine/lysine-rich peptide. Missing residues are prolines or are absent because of exchange with the solvent, dots indicate resonances that disappeared upon binding; the dashed black line indicates the peak intensity decrease due to the titration dilution effect Titrations have been performed at pH6, 20 mM NaCl, 20 mM phosphate buffer. **i** Superposition of ^{1}H-^{15}N HSQC spectra of ^{15}N CXCL12 (0.1 mM) without (black) and with (magenta) arginine/lysine-rich peptide (1:1).

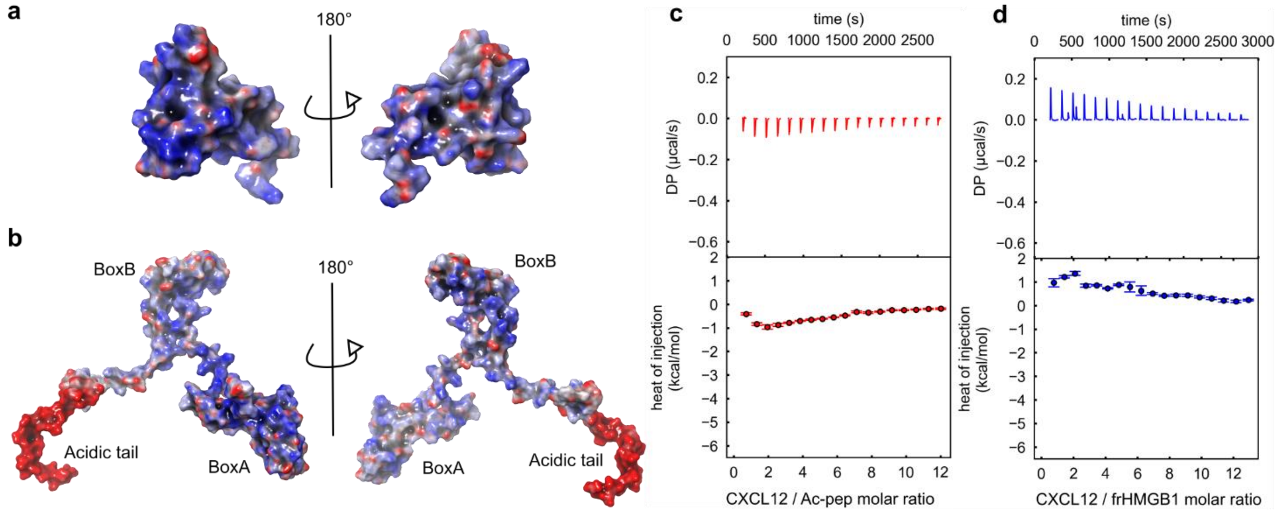

Supplementary Figure 6: Electrostatic interactions drive enthalpic changes in frHMGB1•CXCL12 complex formation. Electrostatic surface representation (as calculated by the Poisson Boltzmann Electrostatic Surface routine in Maestro Schröodinger Suite software (Schrödinger Release 2020-3: Maestro, Schrödinger, LLC, New York, NY, 2021) of **a** CXCL12 and **b** frHMGB1. The ITC sequential heat pulses (upper panel) and the integrated data corrected for heat of dilution (lower panel) of CXCL12 binding to **c** Ac-pep (20 mM TrisHCl at pH 7.5, 150 mM NaCl) and to **d** frHMGB1 (20 mM TrisHCl at pH 7.5, 150 mM NaCl). Data represents peak integration of ITC signal, the error associated with the integrated heat of injection arises from baseline uncertainties and is estimated by the automatic peak integration procedure in NITPIC[3], n=1.

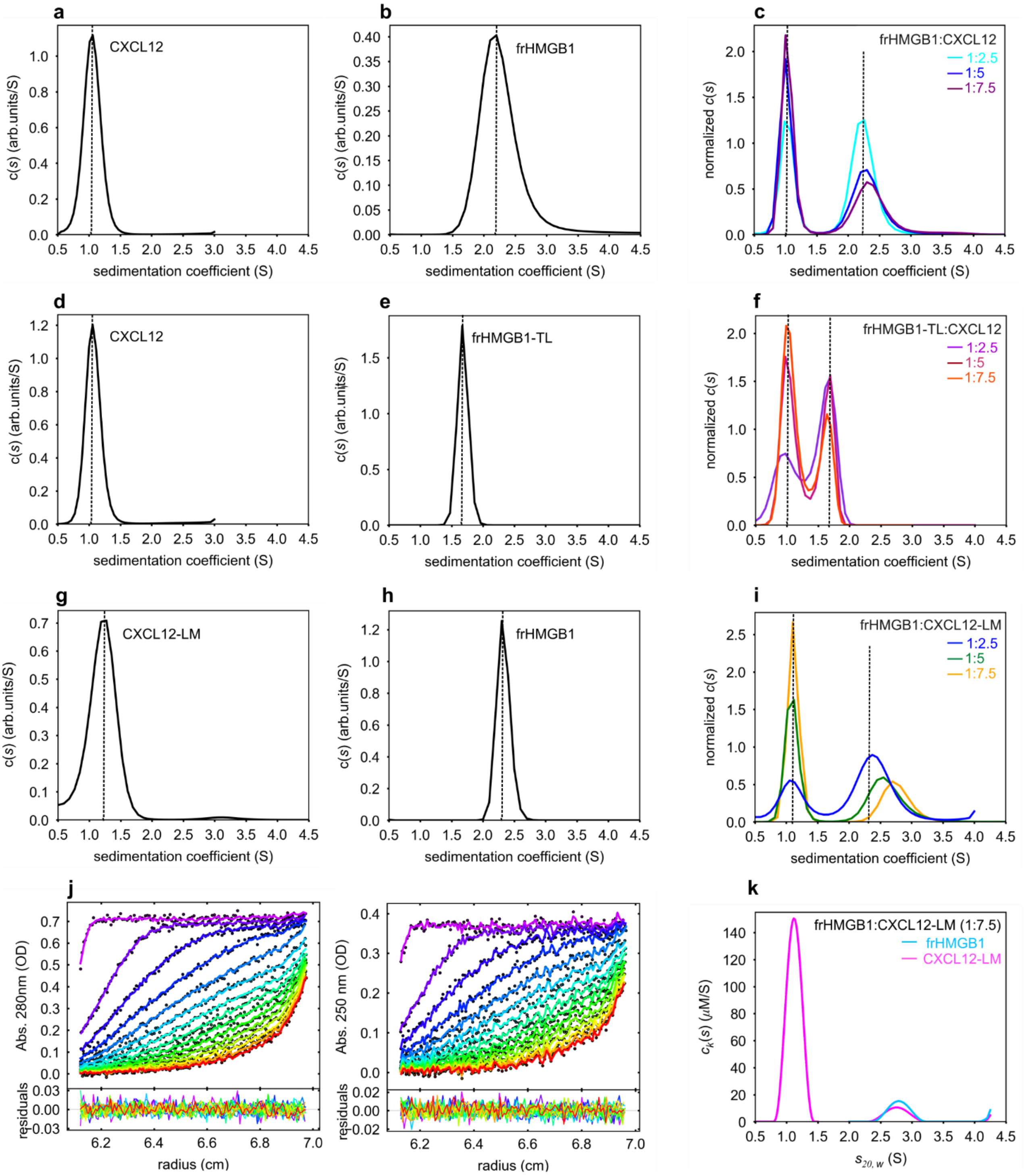

Supplementary Figure 7: Analytical ultracentrifugation analysis of the interaction between CXCL12 and frHMGB1 and frHMGB1-TL and between CXCL12-LM and frHMGB1. Sedimentation coefficient distributions c(*s*) of free **a** CXCL12 (38.2 µM) and **b** frHMGB1 (15.6 µM), scanned by absorbance at 280 nm. **c** Overlay of normalized c(*s*) showing the interaction between frHMGB1 (7.8 µM) and increasing concentrations of CXCL12 (colors). Dotted lines indicate the sedimentation coefficients of the free components. The continuous sedimentation coefficient distribution analysis shows that the peak corresponding to frHMGB1 broadens and shifts to a larger *s* value in an CXCL12 concentration-dependent manner. Experimental conditions: pH 7.5, 20 mM TrisHCl, 150 mM NaCl. Sedimentation coefficient distributions c(*s*) of **d** CXCL12 (38.2 µM) and **e** frHMGB1-TL (15.6 µM), scanned by absorbance at 280 nm. **f** Overlay of normalized c(*s)* showing the interaction between frHMGB1-TL (7.8 µM) and increasing

concentrations of CXCL12 (colors). c(*s*) analysis of the complex shows that the peak corresponding to frHMGB1-TL does not shift to larger c(*s*) values in a CXCL12 concentration-dependent manner, suggesting that the association between the two components is too rapid to be detected in the sedimentation time scale. The dotted lines indicate the sedimentation coefficients of the free components. Experimental conditions: pH 7.5, 20 mM TrisHCl, 50 mM NaCl. Sedimentation coefficient distributions c(*s*) of free **g** CXCL12-LM (37.6 µM) and **h** frHMGB1 (15.6 µM), scanned by absorbance at 280 nm. **i** Overlay of normalized c(*s*) showing the interaction between frHMGB1 (7.8 µM) and increasing concentrations of CXCL12-LM (colors). Dotted lines indicate the sedimentation coefficients of the free components. c(*s*) analysis shows that the peak corresponding to frHMGB1 broadens and shifts to a larger s value in a CXCL12-LM concentration-dependent manner. **j** Global multi-signal sedimentation velocity analysis to determine the stoichiometry of frHMGB1:CXCL12-LM complex, with 7.9 µM frHMGB1 and 51 µM CXCL12. The raw sedimentation signals of the frHMGB1•CXCL12-LM heterocomplex acquired at different time points with absorbance at 280 nm (left), and absorbance at 250 nm (right) with the signal profiles as a function of radius in centimeters. The time-points of the boundaries are indicated in rainbow colors, progressing from purple (early scans) to red (late scans). Only every 3rd data point used in the analysis are shown. Residuals of the fit are shown at the bottom. **k** Decomposition into the component (*k*) sedimentation coefficient distributions, $c_k(s)$ for CXCL12-LM (magenta) and frHMGB1 (cyan line). Integration of sedimentation coefficient distributions of CXCL12-LM and frHMGB1 yields the concentration of the co-sedimenting components in the reaction mixture (Supplementary Table 3).

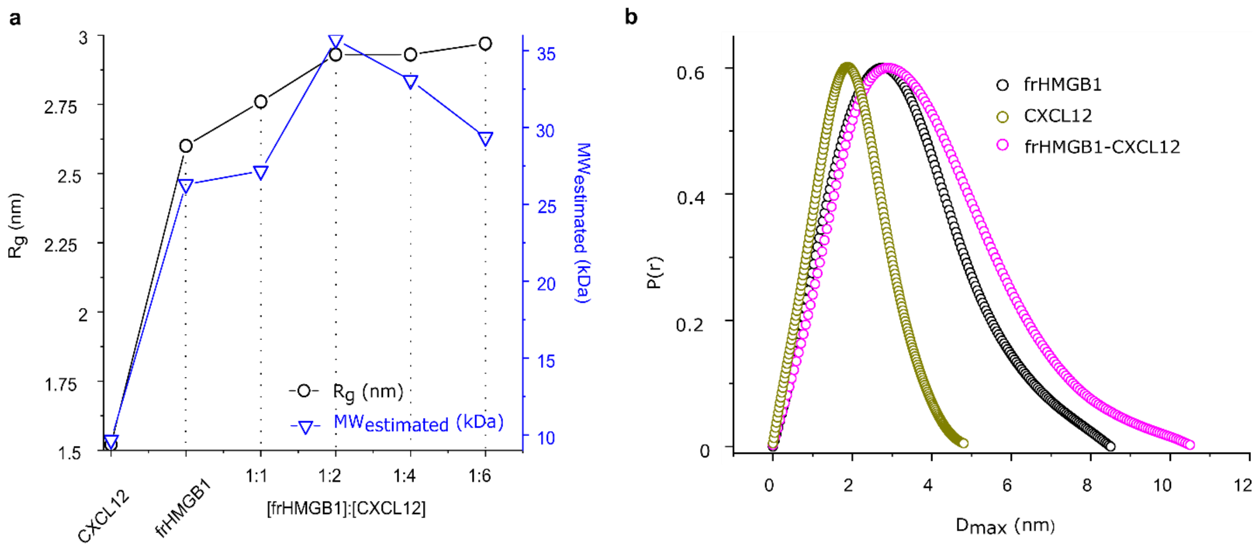

Supplementary Figure 8: Structural parameters of the frHMGB1●CXCL12 complex. a Titration of frHMGB1 with increasing CXCL12 equivalents and estimation of the R_g (in black) and molecular weight (MW, in blue) parameters of the assemblies. At 1:4 and 1:6 conditions a decrease in MW estimation is visible, due to the contribution of free CXCL12. The R_g and MW values are stable at 1:2, suggesting that in this condition the frHMGB1●CXCL12 complex is stable. **b** Distance probability plot with the *P*(*r*) versus D_{max} (in nm) profiles from the SAXS experimental data of CXCL12 (gold circles), frHMGB1 (black circles) and frHMGB1-CXCL12 complex (magenta circles).

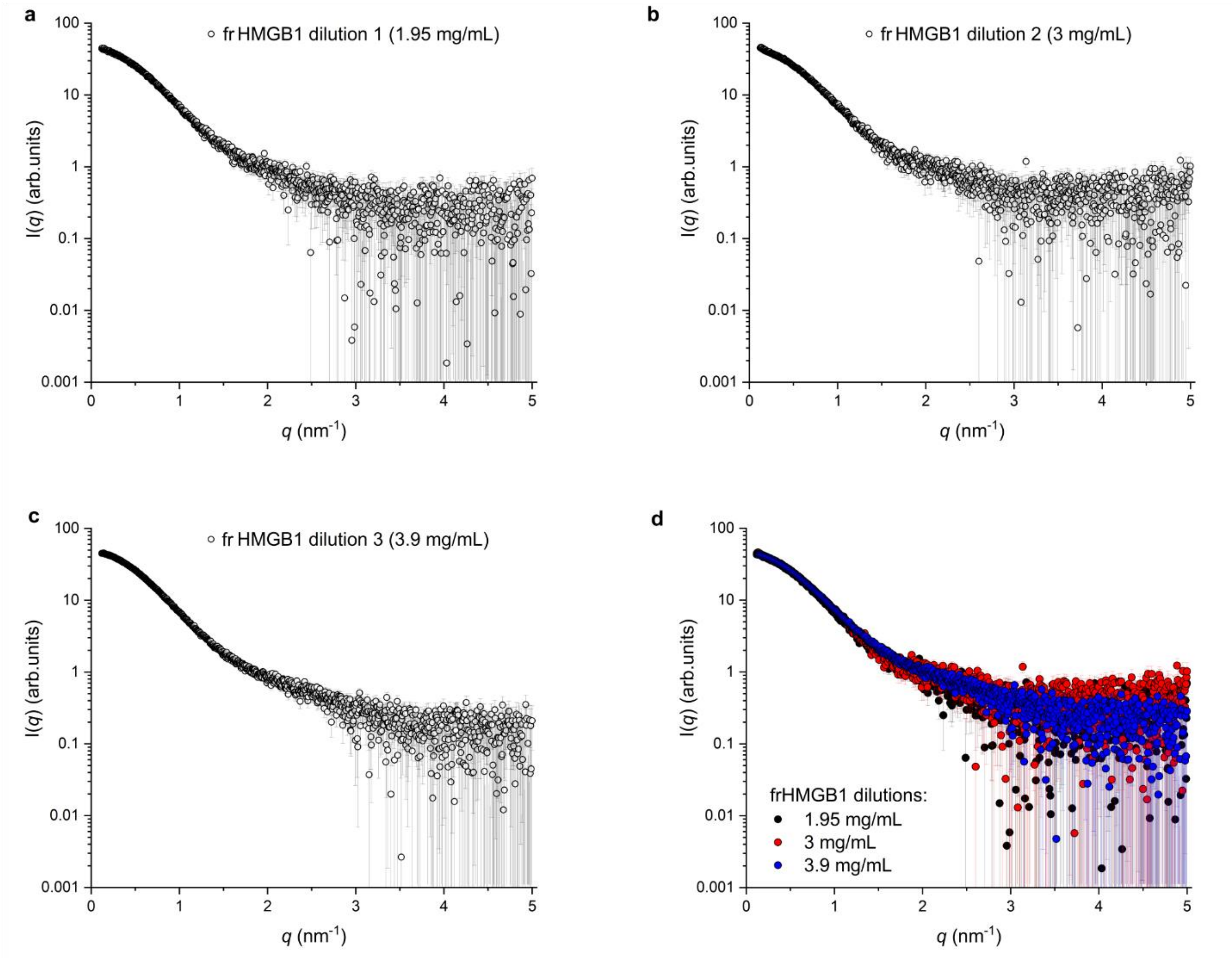

Supplementary figure 9: SAXS dilution series of frHMGB1. Three protein concentrations were tested: **a** 1.95 mg/mL, **b** 3.0 mg/mL and **c** 3.9 mg/mL, **d** superimposition of the three curves at different protein concentrations as indicated in the inset.

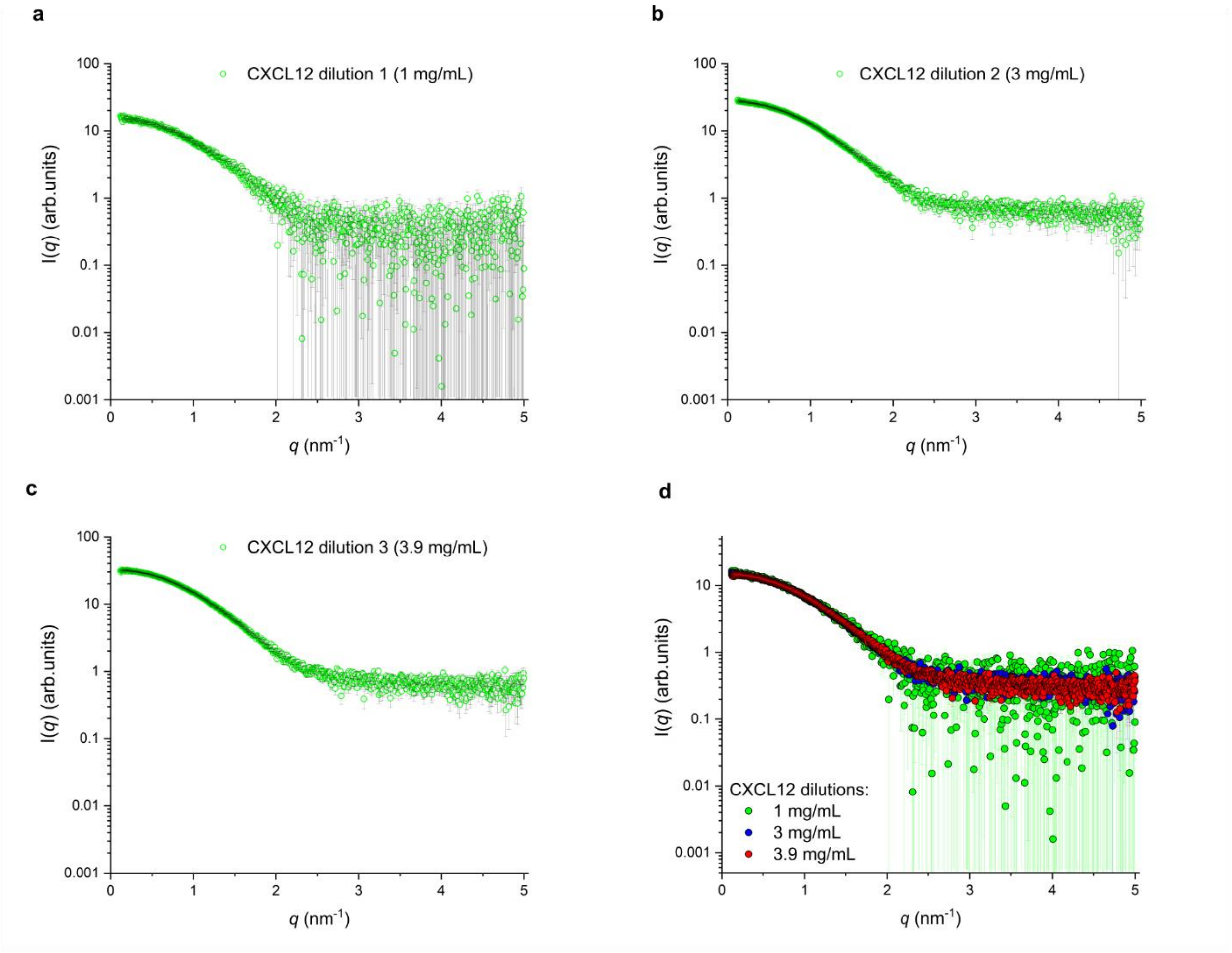

Supplementary figure 10: SAXS dilution series of CXCL12. Three protein concentrations were tested: **a** 1.0 mg/mL, **b** 3.0 mg/mL and **c** 3.9 mg/mL, **d** superimposition of the three curves at different protein concentrations as indicated in the inset.

a

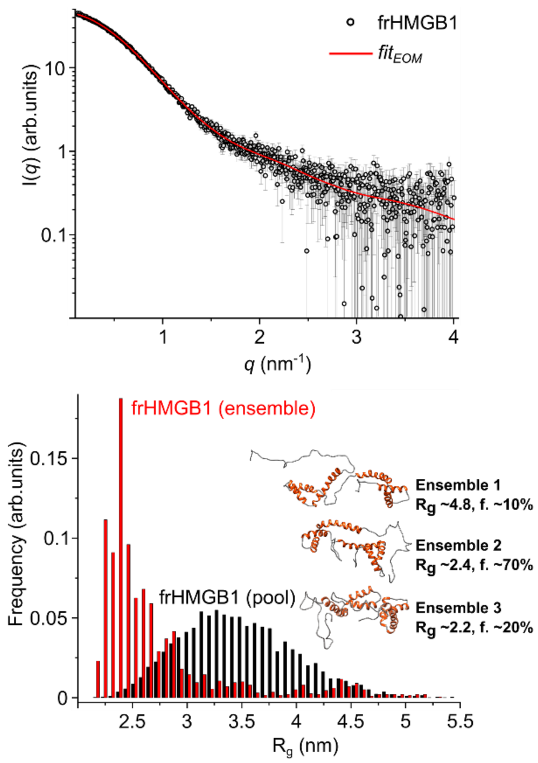

Supplementary figure 11: EOM analysis of free frHMGB1 SAXS data. The EOM algorithm was used to select sub-ensembles of coexisting states from large pools of HMGB1 randomized conformations to fit the SAXS spectrum of frHMGB1. **a** I(*q*) versus *q* experimental SAXS profile for frHMGB1 (black dots) with the EOM fit (red line, χ^2 1.3). The curves are shifted by an arbitrary offset for better comparison. **b** Distribution of the selected ensemble conformers (red bars) and the initial pool of frHMGB1 structures (black bars) as a function of R_g. In the inset representative frHMGB1 structures within the ensemble with the estimated R_g value in nm and the volume fraction of each conformer. EOM conditions: default parameters (10,000 models in initial ensemble, native-like models, constant subtraction allowed); pdb rigid bodies for frHMGB1 BoxA (residues 13-76) and BoxB (residues 99-164) were generated using Alphafold (Uniprot id P63159 https://alphafold.ebi.ac.uk/entry/P63159); position of rigid bodies in the space during simulation: free; segments of frHMGB1 modelled by EOM: 1-12, 77-98, 165-214.

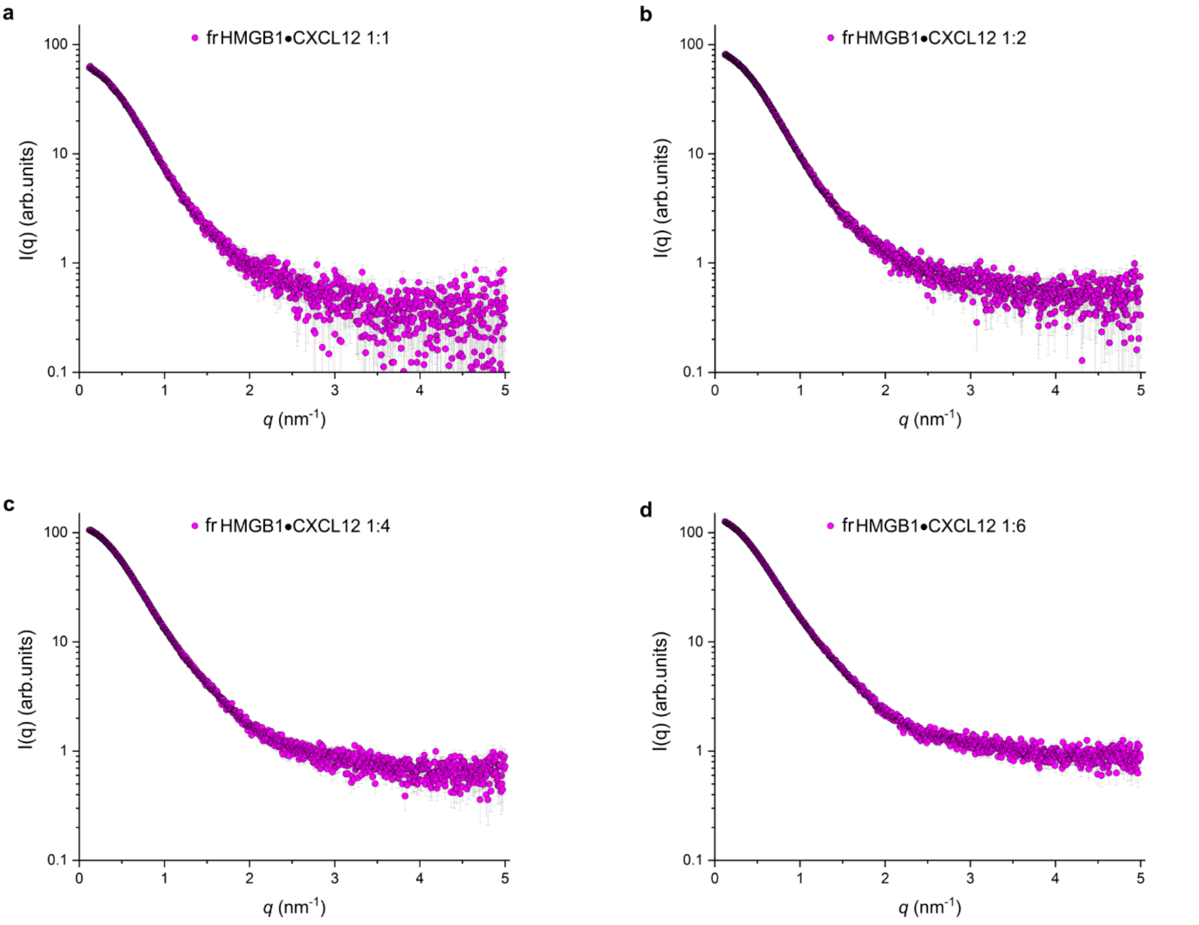

Supplementary Figure 12: SAXS concentration series on frHMGB1 titrated with CXCL12. A range of protein molar ratios were tested: **a** 1:1, **b** 1:2, **c** 1:4, and **d** 1:6 (HMGB1:CXCL12), respectively. The concentration of HMGB1 was consistently maintained at 6.3 μM.

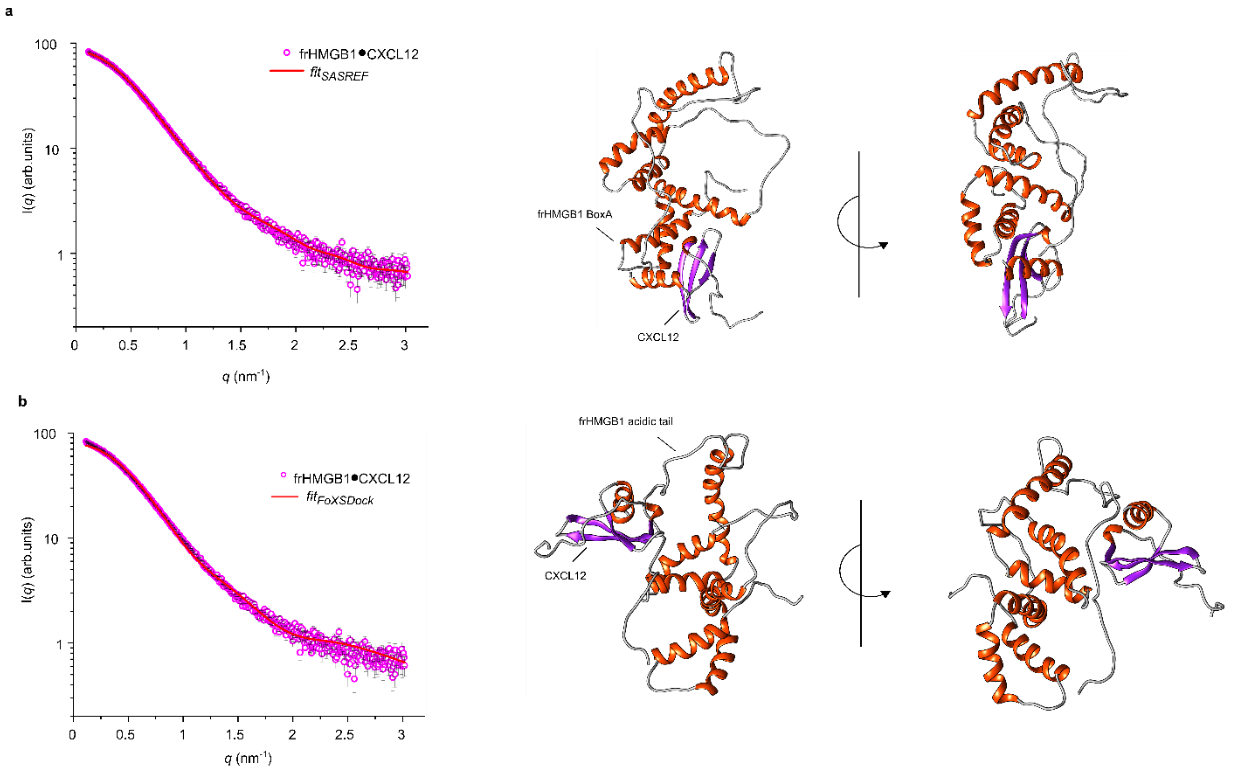

Supplementary Figure 13: SASREF and FoXSDock rigid body modelling of frHMGB1•CXCL12 heterocomplex. a Fits of the SASREF model (red lines, χ^2 value of 1.15) to the experimental data (magenta dots) and the corresponding 3D structural model. **b** FoXSDock model of the complex where CXCL12 binds to the acidic IDR only; here the fitting (red line) shows a χ^2 value of 2.5 indicating that HMGB1 may explore other folding features. On the right the corresponding 3D structural model.

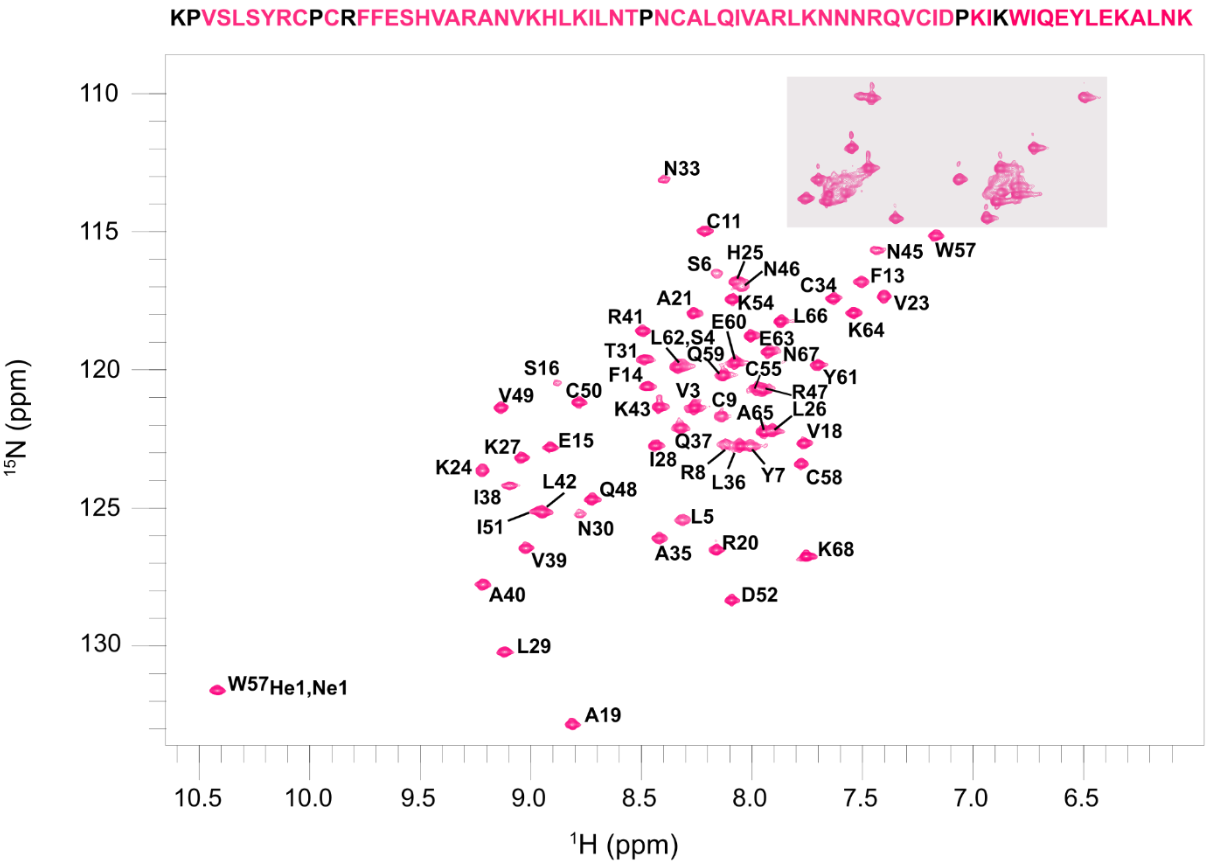

Supplementary Figure 14: ^{1}H-^{15}N HSQC spectrum of ^{15}N CXCL12-LM. Assigned ^{1}H-^{15}N HSQC spectrum of ^{15}N CXCL12-LM (0.1 mM) acquired at a proton resonance frequency of 600.13 MHz, 298 K, in 20 mM NaCl, 20 mM phosphate, pH 6.3. The assigned amides (96% sequence coverage) are colored in pink on the sequence and marked in the spectrum with the amino acid one-letter code and residue number. The grey rectangle indicates the NH_2 groups of glutamines and asparagines.

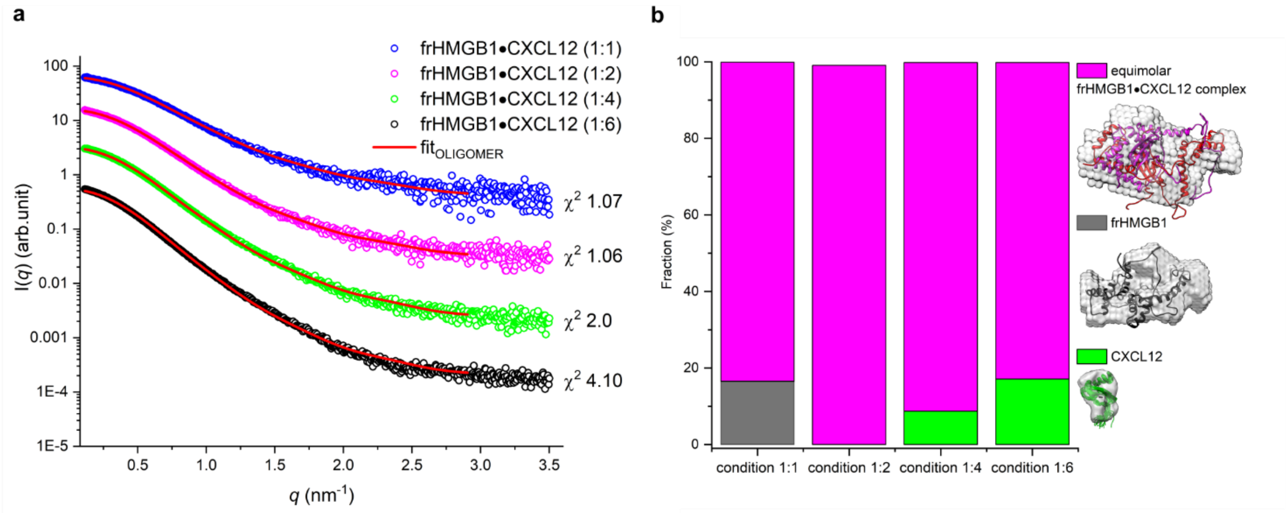

Supplementary Figure 15: Estimation of unbound frHMGB1 and CXCL12 during complex formation. a Fitting with OLIGOMER (red lines, with indicated χ^2 values) to the experimental SAXS data from 1:1 frHMGB1:CXCL12 up to 1:6 molar ratio. **b** Estimation of fraction components within the SAXS data from 1:1 to 1:6 conditions with the contributions of the equimolar frHMGB1•CXCL12 heterocomplex (magenta), free frHMGB1 (grey) and free CXCL12 (green). The best docking complex poses obtained with SASREF and FoXSDock, frHMGB1 structure (one representative structure from EOM), and CXCL12 (pdb: 2KEE, [https://doi.org/10.2210/pdb2KEE/pdb]) have been fitted in their corresponding dummy atoms envelopes.

a

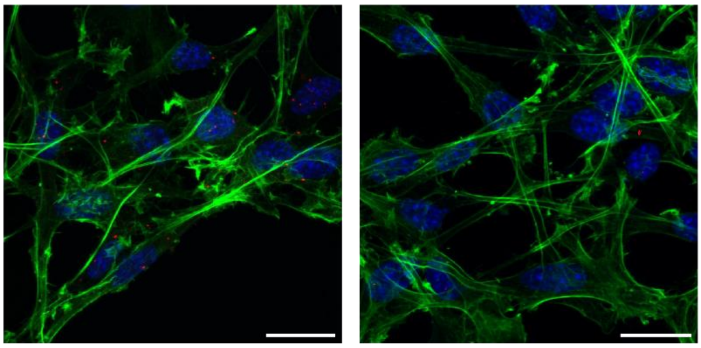

b

Average frHMGB1•CXCL12 dot count	
**Condition**	**Average dots/cell**
**WT:KO (3:1)**	11.6 ± 2.6
**WT:KO (1:3)**	2.9 ± 1.8
**Ratio**	**3.88**

Supplementary Figure 16: Coculture of AB1^{WT} and AB1$^{Cxcr4-/-}$ cells: a To verify whether the PLA signal between frHMGB1•CXCL12 is indeed absent on CXCR4 knockout cells, AB1^{WT} and AB1$^{Cxcr4-/-}$ cells were cocultured at 3:1 (left) and 1:3 (right) ratios. Representative confocal microscopy images of Proximity Ligation Assays (PLAs) performed on the frHMGB1•CXCL12 complex on the surface of cells. The frHMGB1•CXCL12 PLA signal is red, nuclei are blue (Hoechst 33342), and phalloidin is in green. Scale bar; 20 µm. **b** PLA signal was quantified as number of dots per cell in a field of view (FOV) and then calculated as a ratio between the two conditions. Dot counts were 3.88 higher in WT than in KO cells, close to what expected (0.75/0.25=3). Data are presented as mean ± SD; n=4 FOV per condition. The experiment has been repeated twice.

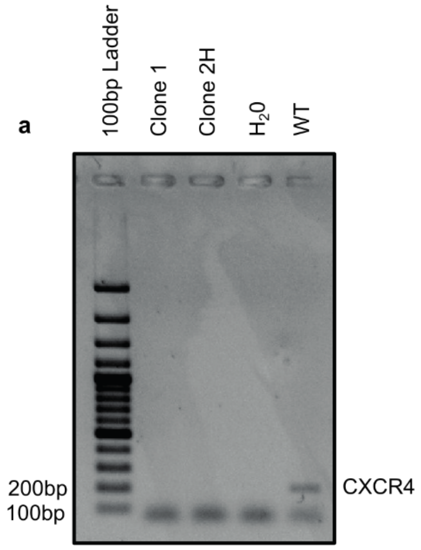

b

qPCR	
Condition	Amplification cycle
AB1^{wt} (CXCR4)	23.90
AB1^{wt} (Beta-actin)	21.04
AB1$^{CXCR4-/-}$ (CXCR4)	34.00
AB1$^{CXCR4-/-}$ (Beta-actin)	20.19

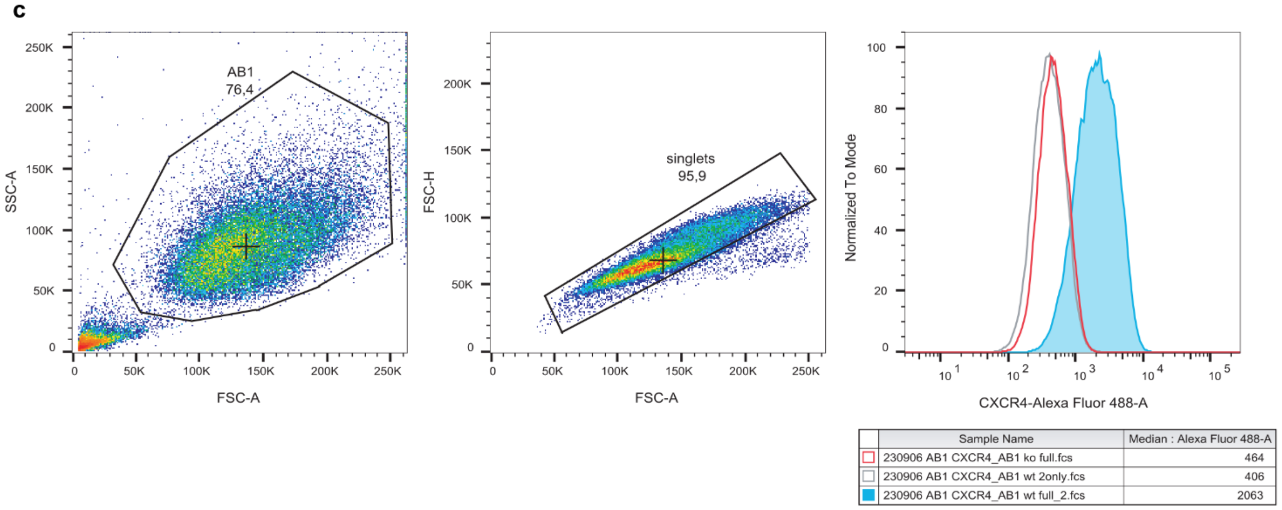

Supplementary Figure 17: *Cxcr4* knockout in AB1 cells. a Knockout of *Cxcr4* in AB1-B/c-LUC cells was verified by GoTaq PCR (clones 1 & 2) against wild-type (WT) AB1 cells (CXCR4 WT: 193bp), and an H_20 blank control. Full uncropped gel can be found in the Source data. **b** A qPCR amplification experiment confirms the knocking out of *Cxcr4*. Sequences for primers used to determine *Cxcr4* knockout are as follows, CXCR4fwd: TTCTCATCCTGGCCTTCA; CXCR4rev: CTGTCATCCCCCTGACTGAT. **c** FACS analysis of AB1$^{Cxcr4-/-}$ cells. After gating the AB1 cell population (left), and selecting single cells (middle), we compared CXCR4 staining of AB1 WT cells (blue shading) with AB1$^{Cxcr4-/-}$ cells (red contour). CXCR4 signal of AB1$^{Cxcr4-/-}$ cells is comparable to that AB1 WT stained only with the secondary antibody (grey contour).

Supplementary Tables

Supplementary Table 1: AUC analysis of CXCL12, frHMGB1 and frHMGB1•CXCL12 at pH 7.5, 20 mM TrisHCl, 50 mM NaCl. Parameters were calculated from c(*s*) model analysis of sedimentation velocity (SV) profile of the free components and from Multi Signal sedimentation velocity (MSSV) model of frHMGB1:CXCL12 (1:7.5).

**SV analysis of single components**		
Parameters	CXCL12	frHMGB1
s[1]	1.1 S	2.3 S
f/f_0[2]	1.3 (globular)	1.4 (elongated)
MW[3]	8.4 kDa	27.1 kDa
RMSD[4]	0.0052	0.0051

**MSSV analysis of frHMGB1:CXCL12 1:7.5**		
Parameters	$CXCL12_{mix}$	$frHMGB1_{mix}$
$s_{20,w}$[5]	2.8 S	3.1 S
$Conc_{exp}$[6]	43.0 µM	7.7 µM
Conc[7]	8.5 µM	7.7 µM
$RMSD_{280nm}$[4]	0.0072	
$RMSD_{250nm}$[4]	0.0050	
D_{norm}[8]	0.08	

[1] sedimentation coefficient expressed in Svedberg (S)
[2] frictional ratio, f is the frictional parameter of the particle and f_0 is the frictional value of a smooth sphere
[3] estimated molecular weight
[4] root mean square deviation of the fitting
[5] sedimentation coefficient normalized to standard solution conditions of water at 20°C
[6] experimental concentration of the components
[7] calculated concentration of the components in the complex sedimentation distribution
[8] determinant of the extinction coefficient matrix

Supplementary Table 2: Calculated values from AUC analysis of CXCL12, frHMGB1 and frHMGB1●CXCL12, pH 7.5, 20 mM TrisHCl,150 mM NaCl. Parameters were calculated from c(*s*) model analysis of sedimentation velocity (SV) profile of the free components and from Multi Signal sedimentation velocity (MSSV) model of frHMGB1:CXCL12 (1:7.5).

**SV analysis of single components**			**MSSV analysis frHMGB1:CXCL12 1:7.5**		
Parameters	CXCL12	frHMGB1	Parameters	$CXCL12_{mix}$	$frHMGB1_{mix}$
s[1]	1.05 S	2.3 S	$s_{(20,w)}$[5]	2.5 S	2.54 S
f/f_0[2]	1.3 (globular)	1.7 (elongated)	$Conc_{exp}$[6]	48.0 μM	7.8 μM
MW[3]	8.6 kDa	34.4 kDa	Conc[7]	6.0 μM	7.6 μM
			$RMSD_{280nm}$[4]	0.0079	
RMSD[4]	0.0056	0.0077	$RMSD_{250nm}$[4]	0.0058	
			D_{norm}[8]	0.09	

[1]sedimentation coefficient expressed in Svedberg (S)
[2]frictional ratio, f is the frictional parameter of the particle and f_0 is the frictional value of a smooth sphere
[3]estimated molecular weight
[4]root mean square deviation of the fitting
[5]sedimentation coefficient normalized to standard solution conditions of water at 20°C
[6]experimental concentration of the components
[7]calculated concentration of the components in the complex sedimentation distribution
[8]determinant of the extinction coefficient matrix

Supplementary Table 3: Calculated values from AUC analysis of CXCL12-LM, frHMGB1 and frHMGB1●CXCL12-LM, pH 7.5, 20 mM TrisHCl, 50 mM NaCl. Parameters were calculated from c(*s*) model analysis of Signal sedimentation velocity (SV) profile of the free components and from Multi Signal sedimentation velocity (MSSV) model of frHMGB1:CXCL12-LM (1:7.5).

**SV analysis of single components**			**MSSV analysis frHMGB1:CXCL12-LM 1:7.5**		
Parameters	CXCL12-LM	frHMGB1	Parameters	$CXCL12\text{-}LM_{mix}$	$frHMGB1_{mix}$
s[1]	1.2 S	1.7 S	$s_{(20,w)}$[5]	2.8 S	2.9 S
f/f_0[2]	1.2 (globular)	1.6 (elongated)	$Conc_{exp}$[6]	51.0 µM	7.9 µM
MW[3]	8.9 kDa	21.1 kDa	Conc[7]	4.9 µM	6.5 µM
			$RMSD_{280nm}$[4]	0.0077	
RMSD[4]	0.0057	0.0049	$RMSD_{250nm}$[4]	0.0057	
			D_{norm}[10]	0.16	

[1]sedimentation coefficient expressed in Svedberg (S)
[2]frictional ratio, f is the frictional parameter of the particle and f_0 is the frictional value of a smooth sphere
[3]estimated molecular weight
[4]root mean square deviation of the fitting
[5]sedimentation coefficient normalized to standard solution conditions of water at 20°C
[6]experimental concentration of the components
[7]calculated concentration of the components in the complex sedimentation distribution
[8]determinant of the extinction coefficient matrix

Supplementary Table 4: Experimental details on SAXS experiments and analysis of CXCL12, frHMGB1 and frHMGB1•CXCL12.

Sample name	**CXCL12**	**frHMGB1**	**frHMGB1-CXCL12**
Organism	*Homo sapiens sapiens*	*Rattus norvegicus*	-
UniProt sequence ID	P48061	P63159	-
Calculated molecular weight (Da)	9183.88	24748.54	33932.42
Total frames (frames used)	10 (8)		
Protein concentration (mg/mL)	1.0, 3.0, 3.9	1.95, 3.0, 3.9	-
Molar ratios (frHMGB1:CXCL12)			1:1, 1:2, 1:4, 1:6
Protein buffer	20 mM Tris pH 7.5, 50 mM NaCl		

Instrument	ESRF BM29
Wavelength (Å)	0.99
q-range (Å)	0.004-0.5
Sample-to-detector distance (m)	2.867
Exposure time	0.5 sec/frame
Temperature (° C)	20
Detector	Pilatus3 X 2M (Dectris)
Flux (photons/s)	2×10^{12}
Beam size (µm)	100 x 100
Sample configuration	1.8 mm quartz glass capillary
Absolute scaling method	Comparison to water in sample capillary
Normalization	To transmitted intensity by beam-stop counter

	**CXCL12**	**frHMGB1**	**frHMGB1-CXCL12**[1]
**Guinier analysis**			
I(0) (cm^{-1})	32.64	43.98	82.69
R_g (nm)	1.52 ± 0.01	2.60 ± 0.02	2.93 ± 0.02
q-range (nm^{-1}), point range	0.035-0.746, 20-140	0.0343-0.27, 13-73	0.0243-0.22, 10-71
***P*(r) analysis**			
I(0) (cm^{-1})	32.8	43.84	83.1
R_g (nm)	1.54	2.62	3.0
D_{max} ($R_{max,}$ nm)	4.8	8.5	10.5
q-range (nm^{-1}), point range	0.035-4.0, 11-752	0.156-3, 13-573	0.0332-2.8, 7-508
Porod volume (nm^3)	19.352	52.729	71.738
χ^2 [total estimate from GNOM]	0.8897	0.8873	0.8573
Mass estimate based on volume (kDa), ratio to predicted	9.676, 1.05	26.36, 1.06	35.82, 1.05
Mass estimate based on Bayesian inference (kDa), ratio to predicted	9.5, 1.03	28.23, 1.1	38.834, 1.1

SAXS data reduction and processing	Dahu and Primus (ATSAS 3.2.1)
Atomic structure modelling	EOM (ATSAS 3.2.1)
3D graphic representation	UCSF Chimera 1.15
Missing sequence modelling	MODELLER 10.1
SAXS-based docking	SASREF (ATSAS 3.2.1), FoXSDock (version main.ec6dbc2)

	CXCL12	frHMGB1	frHMGB1-CXCL12[1]	
EOM: Default parameters, 10,000 models in initial ensemble, native-like models, constant subtraction allowed				
			SASREF	FoXSDock
PDB id for rigid bodies	-	Alphafold P63159	CXCL12: 2KEE<br>frHMGB1: Alphafold P63159	
Rigid bodies (residue numbers)	-	frHMGB1$_{13-76}$<br>frHMGB1$_{99-164}$	CXCL12$_{8-68}$<br>frHMGB1$_{13-76}$<br>frHMGB1$_{99-164}$	CXCL12$_{8-68}$<br>frHMGB1$_{13-76}$<br>frHMGB1$_{99-164}$<br>frHMGB1$_{13-76}$<br>frHMGB1$_{187-205}$
Position of rigid bodies in the space during simulation	-	BoxA (fixed)<br>BoxB (free)	CXCL12 (fixed)<br>BoxA (fixed)<br>BoxB (free)	CXCL12 (fixed)<br>BoxA (free)<br>BoxB (free)<br>acIDR (fixed)
Segments modelled *via* EOM (residue numbers)	-	frHMGB1$_{1-12}$<br>frHMGB1$_{77-98}$<br>frHMGB1$_{165-214}$	CXCL12$_{1-7}$<br>frHMGB1$_{1-12}$<br>frHMGB1$_{77-98}$<br>frHMGB1$_{165-214}$	CXCL12$_{1-7}$<br>frHMGB1$_{1-12}$<br>frHMGB1$_{77-98}$<br>frHMGB1$_{165-186}$<br>frHMGB1$_{206-214}$
χ^2 value for the fitting	-	1.026	0.975	0.979
CORMAP *P*-values[2]	-	0.77	0.422	0.422
Number of representative structures	-	6	5	4
R_{flex} ensemble (%)	-	73.06	69.38	69.16
R_{flex} pool (%)	-	85.40	84.27	85.04
R_{σ} ensemble	-	1.16	0.63	0.72
Weighted average R_g (nm)	-	2.81	2.99	2.95
Weighted average D_{max} (nm)	-	9.07	9.19	9.88
**SASREF**	*Default parameters*			
PDB id for rigid bodies	-		CXCL12: 2KEE<br>frHMGB1: Alphafold P63159	
Contact region; max distance (Å)	-	-	CXCL12$_{23-28}$, frHMGB1$_{34-40}$; 5 Å	
χ^2 value for the fitting	-	-	1.2	
**FoXSDock**	*Default parameters*			
PDB id for rigid bodies	-	-	CXCL12: 2KEE<br>frHMGB1: Alphafold P63159	
χ^2 value for the fitting	-	-	2.5	

	CXCL12	frHMGB1	frHMGB1-CXCL12
**SASBDB ID***	SASDRG9	SASDRH9	SASDRJ9
* https://www.sasbdb.org/project/2028/plawdyohws/			

[1] Parameters were obtained on a sample containing one equivalent of frHMGB1 and two equivalents of CXCL12.

[2.]CORMAP[4] is a correlation based goodness of fit test for assessing differences between one dimensional datasets using only data point correlations over the recorded *q* range or part of it, independently of error estimates. P values are $0.01 \leq P < 0.05$.

Supplementary Methods

Expression and purification of CXCL12, CXCL12-LM, frHMGB1 and frHMGB1-TL

The plasmid of CXCL12 was transformed into *E.coli BL21(DE3)* and cells were grown at 37°C in Luria-Bertani medium. CXCL12 expression was induced by the addition of isopropyl-beta-D-thiolgalactopyranoside (IPTG) at a final concentration of 1 mM when cultures reached an optical density of 2.2 at 600 nm. Induced cultures were grown for an additional 4.5 h at 37°C, harvested by centrifugation at 6,000 g, and stored at −80°C until further processing. The cell pellets were resuspended in 20 ml buffer containing 50 mM TrisHCl (pH 8), 100 mM NaCl, 1 mM EDTA, 5 mM DTT, 0.1 mg DNAse, 0.1 mg RNAse, and 5 mg lysozyme. The resuspended cells were lysed by sonication pulsed 1 s on and 1 s off for 2 min at 60% power. The inclusion bodies were washed twice with buffer containing 50 mM TrisHCl (pH 8), 100 mM NaCl, 1 mM EDTA, and 5 mM DTT, and finally with 50 mM TrisHCl (pH 8), 100 mM NaCl, 1 mM EDTA, 5 mM DTT, and 0.1% Triton X100, followed by solubilization in buffer supplemented with 6 M Guanidinium HCl and 50 mM HEPES pH 6.5 overnight at room temperature. The solubilized fraction was cleared by centrifugation at 17,000 g at 4°C for 30 min and diluted dropwise into refolding buffer containing 50 mM TrisHCl pH 8, 50 mM NaCl, 0.1 mM reduced glutathione, and 0.1 mM oxidized glutathione in a sample:buffer volume ratio of 1:12 and kept overnight at 4°C with stirring. Prior to chromatography, the protein solution was centrifuged at 17,000 g for 30 min. CXCL12 was purified by cation-exchange chromatography using a SP Sepharose resin (SP Sepharose HP, GE Healthcare Bioscience AB, Uppsala, Sweden). The protein was washed with buffer A, supplemented with 50 mM TrisHCl pH 8 and 50 mM NaCl, and eluted with buffer B, containing 50 mM TrisHCl pH 8 and 2 M NaCl. CXCL12 was finally dialyzed against the desired experimental buffer.

For the production of CXCL12-LM, site-directed mutagenesis was performed to introduce mutations L55C and I58C in CXCL12 pET30a expression vector by using standard overlap extension methods. The following oligos were used:

L55C_forward (5'→ 3'): GGTGTGTATTGATCCGAAATGTAAATGGATTCAGGAATACC

L55C_reverse (3' → 5'): GGTATTCCTGAATCCATTTACATTTCGGATCAATACACACC

I58C_forward (5' → 3'): GATCCGAAACTGAAATGGTGTCAGGAATACCTGGAAAAAGC

I58C_reverse (3' → 5'): GCTTTTTCCAGGTATTCCTGACACCATTTCAGTTTCGGATC

CXCL12-LM was then expressed and purified as described for CXCL12.

Uniformly ^{15}N- and ^{13}C-^{15}N-labeled CXCL12 and CXCL12-LM were expressed by growing *E. coli BL21 (DE3)* cells in minimal bacterial medium containing $^{15}NH_4Cl$, with or without ^{13}C-D-glucose as sole nitrogen and carbon sources.

frHMGB1 and frHMGB1-TL were expressed in *pLys BL21 (DE3) E. coli* cells. Cells were grown at 37°C until the optical density at 600 nm reached 0.8 absorbance units. Gene expression was induced by the addition of isopropyl b-D-1-thiogalactopyranoside (IPTG) to a final concentration of 1 mM. After 18 h of incubation at 25°C with shaking, cells were harvested by centrifugation. The cells were resuspended in lysis buffer (20 mM Tris–HCl pH 8.0, 150 mM NaCl, 10 mM imidazole, 2 mM b-mercaptoethanol, 0.2% NP-40, Complete EDTA-free protease inhibitor (Roche), 2 μg/ml DNase, 20 μg/ml RNase, 1mg/ml lysozyme) and lysed by sonication. Cell debris was removed by centrifugation at 11,000 g for 45 min at 4°C. The soluble 6His-tagged proteins were purified from the supernatant by affinity chromatography using Ni^{2+}-NTA agarose resin (Qiagen, Hilden, Germany). After several washing steps, proteins were eluted in 20 mM Tris pH 8.0, 150 mM NaCl, 300 mM imidazole pH8, and 2 mM b-mercaptoethanol. The 6His-tag was removed by overnight incubation at 4°C with TEV protease. During incubation, the sample was dialyzed against 20 mM Tris pH 8.0, 150 mM NaCl, 2 mM b-mercaptoethanol. Uncleaved 6His-tagged protein and TEV protease were then removed by repassing the sample over Ni^{2+}-NTA resin. frHMGB1 was further purified on a HitrapQ ion-exchange column using buffer A (20 mM Tris pH 8, 25-50 mM NaCl, 2 mM b-mercaptoethanol) and buffer B (20 mM Tris pH 8, 1 M NaCl, 2 mM b-mercaptoethanol) to create a linear gradient of NaCl. frHMGB1 elutes at 500 mM NaCl. frHMGB1-TL was purified by gel filtration on a Superdex-75 column (Amersham Biosciences, Milan, Italy) equilibrated in 20 mM phosphate buffer (pH 7.3), 150 mM NaCl, and 1 mM dithiothreitol (DTT). For MST experiments, the 6His-tag was not removed. Uniformly ^{15}N labeled samples were prepared using M9 minimal bacterial growth media appropriately supplemented with $^{15}NH_4Cl$.

OLIGOMER Analysis

To estimate the volume fractions of the individual components contributing to the overall scattering signal we utilized OLIGOMER from the ATSAS 3.1.4 package[1]. OLIGOMER uses form factors to fit experimental scattering data from multi-component protein mixtures. On turn, form factors can be derived from known structures or from dummy

atoms models that can be computed using the ATSAS 3.1.4 program CRYSOL[1]. Because of the flexibility of both frHMGB1 and frHMGB1●CXCL12 we used the dummy atoms envelopes. The pair distance distribution functions, P(*r*), of CXCL12, frHMGB1 and frHMGB1●CXCL12 were used to calculate *ab initio* models in P1 symmetry with DAMMIF (ATSAS 3.1.4)[2]. Both the free frHMGB1 structure (one representative structure from EOM) and the best docking poses obtained with SASREF (within ATSAS 3.1.4) and FoXSDock (version main.ec6dbc2) fit relatively well in the envelopes obtained and are in line with the experimental R_g and D_{max} (Supplementary Figure 15a).

According to OLIGOMER analysis, when frHMGB1 is titrated with one equivalent of CXCL12 an equimolar frHMGB1•CXCL12 heterocomplex is formed representing about 80% of the total volume fraction, alongside a non-negligible fraction of free frHMGB1 in solution (approximately 18%), which contributes to the overall scattering curve. In this condition the volume fraction of CXCL12 appears negligible. In the presence of two equivalents of CXCL12, the equimolar heterocomplex constitutes the predominant volume fraction within the SAXS spectrum, with virtually no contribution of free frHMGB1 and CXCL12 to the volume fraction (Supplementary Figure 15b). Conversely, at 1:4 and 1:6 ratios OLIGOMER detects an increased presence of free CXCL12, approximately representing 7% and 18% of the total volume fractions, respectively.
Taken together, the OLIGOMER estimation of the contributions to the scattering curves of the different components supports the choice to analyse the 1:2 condition as the one that best represents the scattering behaviour of frHMGB1●CXCL12 in solution.

Supplementary References

1. Manalastas-Cantos, K. *et al.* ATSAS 3.0: Expanded functionality and new tools for small-angle scattering data analysis. *J. Appl. Crystallogr.* **54**, 343–355 (2021).

2. Franke, D. & Svergun, D. I. DAMMIF, a program for rapid ab-initio shape determination in small-angle scattering. *J. Appl. Crystallogr.* **42**, 342–346 (2009).

3. Keller, S. *et al.* High-precision isothermal titration calorimetry with automated peak-shape analysis. *Anal. Chem.* **84**, 5066–73. (2012).

4. Franke, D., Jeffries, C. M. & Svergun, D. I. Correlation Map, a goodness-of-fit test for one-dimensional X-ray scattering spectra. *Nat. Methods* **12**, (2015).
