# Supplementary material for: The acidic intrinsically disordered region of the inflammatory mediator HMGB1 mediates fuzzy interactions with CXCL12
Source: Nat Commun. 2024 Feb 8;15:1201. doi: 10.1038/s41467-024-45505-7 (PMC10853541; doi:10.1038/s41467-024-45505-7)
Supplement: Supplementary file 3 — Reporting Summary [file 41467_2024_45505_MOESM3_ESM.pdf]

nature portfolio

Corresponding author(s): Giovanna Musco and Michela Ghitti

Last updated by author(s): 01/18/2024

Reporting Summary

Nature Portfolio wishes to improve the reproducibility of the work that we publish. This form provides structure for consistency and transparency in reporting. For further information on Nature Portfolio policies, see our Editorial Policies and the Editorial Policy Checklist.

Statistics

For all statistical analyses, confirm that the following items are present in the figure legend, table legend, main text, or Methods section.

n/a	Confirmed	
☐	☒	The exact sample size (n) for each experimental group/condition, given as a discrete number and unit of measurement
☐	☒	A statement on whether measurements were taken from distinct samples or whether the same sample was measured repeatedly
☐	☒	The statistical test(s) used AND whether they are one- or two-sided<br>*Only common tests should be described solely by name; describe more complex techniques in the Methods section.*
☒	☐	A description of all covariates tested
☒	☐	A description of any assumptions or corrections, such as tests of normality and adjustment for multiple comparisons
☐	☒	A full description of the statistical parameters including central tendency (e.g. means) or other basic estimates (e.g. regression coefficient) AND variation (e.g. standard deviation) or associated estimates of uncertainty (e.g. confidence intervals)
☐	☒	For null hypothesis testing, the test statistic (e.g. F, t, r) with confidence intervals, effect sizes, degrees of freedom and P value noted<br>*Give P values as exact values whenever suitable.*
☒	☐	For Bayesian analysis, information on the choice of priors and Markov chain Monte Carlo settings
☒	☐	For hierarchical and complex designs, identification of the appropriate level for tests and full reporting of outcomes
☒	☐	Estimates of effect sizes (e.g. Cohen's d, Pearson's r), indicating how they were calculated

Our web collection on statistics for biologists contains articles on many of the points above.

Software and code

Policy information about availability of computer code

Data collection	NMR: Topspin 3.2. Bruker,<br>ITC: MicroCal PEAQ-ITC (Malvern)<br>MST: NanoTemper® Monolith NT.115,<br>SAXS data collection: ESRF bioSAXS beamline BM29, Grenoble, France at a detector distance of 2.869 m pyFAI and BsxCuBE ;<br>Micrograph: Leica TCS SP5 X confocal microscope (Leica Biosystems)<br>FACS : BD FACScantoii, BD Biosciences

Data analysis	NMR:Topspin 3.2, CCPNmr Analysis 2.4, NMRpipe Version 10.9, TITAN (v1.2), xmgrace (v 5.1.25),<br>MST: NanoTemper Analysis software v2.3.<br>ITC: MicroCal PEAQ-ITC Analysis Software v.1.4<br>AUC and ITC: SEDNTERP 1.0, SEDFIT 16.1c, NITPIC 2.0.0, SEDPHAT 15.2b, GUSSI 1.4.2 ;<br>SAXS: PRIMUS, DAMMIF, SASREF, OLIGOMER and CORMAP within ATSAS 3.2.1. GNOM version 5.0 (r14886), MODELLER 10.1, FoXDocks, version main.ec6dbc2<br>SAXS data graphical representation: OriginPro (Version 2022. OriginLab Corporation, Northampton, MA, USA).<br>PYMOL, Molecular Graphics System, open source version, Schrödinger, LLC and UCSF Chimera 1.16;<br>PLA: Python 3.10 to run Cellpose, ImageJ/Fiji, GraphPad Prism, version 8.4.0 used for the generation of original graphs and statistical tests.<br>Figures assembly: Inkscape v. 0.92

For manuscripts utilizing custom algorithms or software that are central to the research but not yet described in published literature, software must be made available to editors and reviewers. We strongly encourage code deposition in a community repository (e.g. GitHub). See the Nature Portfolio guidelines for submitting code & software for further information.

Data

Policy information about availability of data

All manuscripts must include a data availability statement. This statement should provide the following information, where applicable:

- Accession codes, unique identifiers, or web links for publicly available datasets
- A description of any restrictions on data availability
- For clinical datasets or third party data, please ensure that the statement adheres to our policy

All SAXS data have been deposited to the SASBDB data bank with the codes:
SASDRH9 (https://www.sasbdb.org/data/SASDRH9/);
SASDRG9 (https://www.sasbdb.org/data/SASDRG9/);
SASDRJ9 (https://www.sasbdb.org/data/SASDRJ9/)
NMR backbone assignments for CXCL12-LM used in this study are available under BMRB entry ID 52209 (https://bmrb.io/data_library/summary/index.php?bmrbId=522099).
NMR backbone assignments of HMGB1, HMGB1-TL, CXCL12 are available under BMRB entry ID 15149 (https://bmrb.io/data_library/summary/index.php?bmrbId=15149) ID 15148 (https://bmrb.io/data_library/summary/index.php?bmrbId=15148) and ID 16519 , (https://bmrb.io/data_library/summary/index.php?bmrbId=16519), respectively.
The NMR Coordinates of CXCL12, CXCL12-LM, and HMGB1-TL are available in the PDB with the following accession codes 2KEE (https://doi.org/10.2210/pdb2KEE/pdb), 2N55 (https://doi.org/10.2210/pdb2N55/pdb); 2YRQ (https://doi.org/10.2210/pdb2YRQ/pdb), respectively.
The crystallographic structure of CXCL12 with two monomers in the asymmetric unit is available in the PDB with the code 1QG7 (https://doi.org/10.2210/pdb1QG7/pdb).
The alphafold model of HMGB1 is available in the AlphaFold Protein Structure Database (entry: AF-P63159 (https://alphafold.ebi.ac.uk/entry/P63159 .
Source data are provided as Source Data file with this paper and can be also found in https://doi.org/10.6084/m9.figshare.24574522. All other data are available from the authors upon request.

Human research participants

Policy information about studies involving human research participants and Sex and Gender in Research.

Reporting on sex and gender	n/a
Population characteristics	n/a
Recruitment	n/a
Ethics oversight	n/a

Note that full information on the approval of the study protocol must also be provided in the manuscript.

Field-specific reporting

Please select the one below that is the best fit for your research. If you are not sure, read the appropriate sections before making your selection.

☒ Life sciences ☐ Behavioural & social sciences ☐ Ecological, evolutionary & environmental sciences

For a reference copy of the document with all sections, see nature.com/documents/nr-reporting-summary-flat.pdf

Life sciences study design

All studies must disclose on these points even when the disclosure is negative.

Sample size	No experiments requiring sample size were used in this study.<br>Where needed experiments were repeated at least three times for statistical analysis.<br>For PLA experiments performed with AB1 cells 20000 cells per slide were seeded so that the cells were not confluent, to allow for the PLA signal to be clearly distinguished between cells.
Data exclusions	In MST titration of CXCL12 with HMGB1 at 20 mM NaCl and of Ac-pep with CXCL12 at 150 mM the first point at highest ligand concentration was excluded because it was considered as outlier
Replication	If applicable we included the number of independent replicas.<br>ITC and MST have been performed two and three times, respectively.<br>For each buffer condition AUC experiments were performed once yielding similar reproducible results. Fitting analysis has been performed at least three times to verify convergence<br>SAXS spectrum was derived from 10 technical replicates at three different concentrations<br>HSQC experiments were averaged over at least 4 independent transients (scans).
Randomization	Randomization was not necessary, because all experimental parameters were precisely controlled and the experiments were performed with standardized ingredients. Randomization is typically not used in this field
Blinding	Investigators were not blinded. Blinding during data collection was not needed because all experimental conditions were precisely controlled.

Reporting for specific materials, systems and methods

We require information from authors about some types of materials, experimental systems and methods used in many studies. Here, indicate whether each material, system or method listed is relevant to your study. If you are not sure if a list item applies to your research, read the appropriate section before selecting a response.

Materials & experimental systems

n/a	Involved in the study	
☐	☒	Antibodies
☐	☒	Eukaryotic cell lines
☒	☐	Palaeontology and archaeology
☒	☐	Animals and other organisms
☒	☐	Clinical data
☒	☐	Dual use research of concern

Methods

n/a	Involved in the study	
☒	☐	ChIP-seq
☐	☒	Flow cytometry
☒	☐	MRI-based neuroimaging

Antibodies

Antibodies used	Primary antibodies: Mouse monoclonal anti-HMGB1 (HMGBiotech, #HM-901); goat polyclonal anti-CXCL12 (R&D Systems, #AF-310-NA). Secondary antibodies: anti-mouse PLUS secondary antibody (Duolink, Sigma-Aldrich, #DUO92001); anti-goat MINUS secondary antibody. Conjugated antibodies: Phalloidin-FITC (Sigma-Aldrich, #P5282)
Validation	For validation see:<br>Anti-HMGB1: https://www.hmgbiotech.eu/wp-content/uploads/2016/04/901.pdf<br>Anti-CXCL12: https://resources.rndsystems.com/pdfs/datasheets/af-310-na.pdf?v=20240110&_ga=2.72364115.1336734236.1704885742-606810628.1704885742<br>Anti-mouse PLUS: https://www.sigmaaldrich.com/certificates/sapfs/PROD/sap/certificate_pdfs/COFA/Q14/DUO92001-100RXN0000282941.pdf<br>Anti-goat MINUS: https://www.sigmaaldrich.com/certificates/sapfs/PROD/sap/certificate_pdfs/COFA/Q14/DUO92006-30RXN0000287126.pdf<br>Phalloidin-FITC: https://www.sigmaaldrich.com/certificates/COFA/P5/P5282/P5282-.1MG________SLBV4340__.pdf

Eukaryotic cell lines

Policy information about cell lines and Sex and Gender in Research

Cell line source(s)	AB1 mouse malignant mesothelioma cells (MM; Cell Bank, Australia), cell bank catalogue number: #CBA-0144. This cell line was originally isolated and characterized by Davis et al., Int. J. Cancer (1992; DOI: https://doi.org/10.1002/ijc.2910520609), and then distributed by Cell Bank, Australia. With these AB1 cells, we also generated a AB1 Cxcr4 knockout cell line.
Authentication	The cell line is authenticated by Cell Bank, Australia. See PDF document of the product datasheet entitled “AB1 MURINE MALIGNANT MESOTHELIOMA CELLS from ECACC”. This cell line was originally isolated and characterized by Davis et al., Int. J. Cancer (1992; DOI: https://doi.org/10.1002/ijc.2910520609).<br><br>For AB1 Cxcr4 knockout cells, we describe how we generated the knockout cells in the methods section “Generation of Cxcr4 knockout AB1 cells”. We authenticated these knockout cells using, Taqman DNA PCR, RT-qPCR, and FACS analysis. All of which is provided within Supplementary figure 17 of this manuscript.
Mycoplasma contamination	Mycoplasma free confirmed by mycoblue mycoplasma detector kit (Vazyme)
Commonly misidentified lines (See ICLAC register)	AB1 are not commonly misidentified

Flow Cytometry

Plots

Confirm that:

- [x] The axis labels state the marker and fluorochrome used (e.g. CD4-FITC).
- [x] The axis scales are clearly visible. Include numbers along axes only for bottom left plot of group (a 'group' is an analysis of identical markers).
- [x] All plots are contour plots with outliers or pseudocolor plots.
- [x] A numerical value for number of cells or percentage (with statistics) is provided.

Methodology

Sample preparation	AB1 cells, both wild-type and Cxcr4 knockout, were harvested from cell culture and stained for CXCR4 only.
Instrument	BDCantoii, BD Biosciences
Software	BDFACSDiva software was used for sample acquisition and FlowJo was used for data analysis
Cell population abundance	N/A
Gating strategy	The gating strategy is provided in Supplementary figure 17. The initial gating was performed on FSC and SSC.

- [x] Tick this box to confirm that a figure exemplifying the gating strategy is provided in the Supplementary Information.
